# The Involvement of Energy Metabolism and Lipid Peroxidation in Lignin Accumulation of Postharvest Pumelos

**DOI:** 10.3390/membranes10100269

**Published:** 2020-09-30

**Authors:** Huiling Yan, Junjia Chen, Juan Liu

**Affiliations:** 1 Guangdong Engineering Lab of High Value Utilization of Biomass, Institute of Bioengineering, Guangdong Academy of Sciences, Guangzhou 510316, China; gzcsircjj@163.com; 2 Key Laboratory of Plant Resources Conservation and Sustainable Utilization, Guangdong Provincial Key Laboratory of Applied Botany, South China Botanical Garden, Chinese Academy of Sciences, Guangzhou 510650, China; hlingyan@scbg.ac.cn; 3 South China Botanical Garden, Chinese Academy of Sciences, Beijing 100049, China

**Keywords:** pumelo, lignin, energy, ROS, lipid peroxidation, membrane integrity

## Abstract

Lignification is especially prominent in postharvest pumelo fruit, which greatly impairs their attractiveness and commercial value. This study investigated the energy metabolism and lipid peroxidation and their relationship with accumulated lignin content in juice sacs of “Hongroumiyou” (HR) during 90 d of storage at 25 °C. The results indicated that, the alterations of energy metabolism in juice of sacs of postharvest pumelos was featured by a continuous decline in energy charge and ATP/ADP; an increase in succinic dehydrogenase (SDH) activity before 30 d and increases in activities of cytochrome c oxidase (CCO) and F_0_F_1_-ATPase before 60 d; but declines in activities of Ca^2+^-ATPase and H^+^-ATPase. Additionally, enhanced contents of H_2_O_2_, O_2_^−^, and –OH scavenging rate; increased malondialdehyde (MDA) content; and transformation of unsaturated fatty acids (USFA) to saturated fatty acids (USFA) and reduced USFA/SFA (U/S) could result in lipid peroxidation and membrane integrity loss. Moreover, correlation analysis showed that lignin accumulation was in close relation to energy metabolism and lipid peroxidation in juice sacs of postharvest pumelos. These results gave evident credence for the involvement of energy metabolism and lipid peroxidation in the lignin accumulation of HR pumelo fruit during postharvest storage.

## 1. Introduction

Pumelo (*Citrus maxima* (Burm.) Merr.), a citrus fruit of the family *Rutaceae*, is native to Asia and cultivated largely in China, Southeast Asia, and southern Japan [1]. Pumelo fruit is widely consumed because of its unique fragrance, good nutrition, and storability [2,3]. However, improper storage could promote the occurrence of lignin accumulation, which consequently result in quality deterioration and commodity loss of postharvest pumelo fruit [4,5,6]. Our previous study illustrated that the sucrose metabolism and ATP deficit contributed to the accumulated lignin content in harvested pumelos during storage [6]. Nevertheless, this is just an iceberg of the complex mechanism of lignification process during postharvest pumelo fruit storage. Therefore, to further explore the main factors affecting the lignin accumulation is important in controlling the quality deterioration of postharvest pumelo fruit.

Energy metabolism, which plays a role in postharvest crops, has been extensively reported [7,8,9,10]. Specifically, energy status was important in regulating senescence, ripening, biotic, and abiotic stress tolerance of postharvest horticulture crops [11,12,13,14,15,16,17]. It was shown that the deterioration of the quality and occurrence of disease in postharvest crops such as longan, litchi, strawberry, loquat, orange, and pear, were closely related to low energy status [18,19,20,21,22]. Other studies showed that sufficient ATP supplying by exogenous treatments could extend shelf life [23], maintain quality [9,24], and ameliorate stresses [25]. It was well documented that energy deficit highly contributed to lignification of loquat fruit [26], water bamboo shoot [27], and mushroom [28] during postharvest storage. Moreover, the possible link between energy deficit and lignification was reinforced by the observation that declined ATP level accelerated the lignification process of three postharvest pumelo cultivars in our previous work [6]. Energy charge (EC) could reflect cellular energy status [18], which is closely influenced by some critical enzymes, such as cytochrome *c* oxidase (CCO), succinic dehydrogenase (SDH), ATPase, nicotinamide adenine dinucleotide phosphate (NADP), and nicotinamide adenine dinucleotide (NAD). ATPase plays a pivotal role in regulating substance transport, ATP synthesis, and cellular homeostasis [7,18,29]. F_0_F_1_-ATPase is critical in providing energy via oxidative phosphorylation and converting the energy of protons transported across membrane to synthesize ATP [30]. A previous study reported that changes in activities of energy-related enzymes, including CCO, SDH, and ATPase, are correlative to the energy status of plant tissues [31]. SDH is a key enzyme in the respiratory chain, which is responsible for reducing ubiquinone (Q)^1^ to succinate [32]. CCO is a terminal enzyme responsible for catalyzing the transport of electrons to O_2_, where proton gradient formed to promote ATP synthesis [33,34]. Therefore, SDH and CCO were considered as markers in the respiratory chain [35]. Quality deterioration during postharvest storage is usually accompanied by reduced ATP level, changes in activities of energy metabolism-involved enzymes and contents of NAD and NADP in various postharvest crops, such as pear, broccoli, longan, and litchi [9,18,19,29,36]. However, the importance of energy deficit for promotion of lignin accumulation has been analyzed in our previous work, while the participation of key enzymes in that lignification process of postharvest pumelo fruit is still not yet elucidated.

Fruit ripening and senescence is accompanied by disorder of ROS metabolism, resulting in excessive accumulation of ROS and malondialdehyde (MDA), which result in continuous lipid peroxidation and membrane injury, which consequently accelerate the quality deterioration of postharvest longan fruit [37], kiwifruit [38], pineapple [39], mushroom [40], and litchi [41]. The role of lipid metabolism on texture deterioration and senescence processes has been well reported, however, whether this role is related to lignin accumulation is not yet elucidated. It is worth mentioning that lignin biosynthesis initiates with phenylpropanoid pathway to produce monolignols within plant cells and ends with their radical polymerization via the peroxidases and laccases which distribute in the apoplastic space within the plant cell wall, and this implies that monolignols must cross the cell membrane before incorporation into the growing lignin polymer [42,43,44]. Moreover, monolignols are transported passively and membrane translocation rates can be controlled by the delivery and utilization rates and membrane concentration gradients [42]. Therefore, it could be inferred that the membrane integrity loss could initiate decompartmentalization of monolignols and polymerization-related enzymes, finally promote the process of lignin biosynthesis. However, previous studies mainly focus on the role of ROS metabolism in the activities of lignin biosynthesis-related enzymes [6,45], while that of membrane destruction caused by lipid peroxidation in the lignin biosynthesis have been scarcely reported to date. Therefore, there is a critical need for further investigation on lignin accumulation in association with energy metabolism and lipid peroxidation in postharvest pumelos.

The current study aimed to illustrate the relationship of energy metabolism and lipid peroxidation with lignin accumulation in juice sacs of postharvest “Hongroumiyou” (HR) pumelos during storage. The EC, ATP/ADP, levels of NAD and NADP, activities of CCO, SDH, F_0_F_1_-ATPase, H^+^-ATPase and Ca^2+^-ATPase, contents of H_2_O_2_, O_2_^−^, and MDA, –OH scavenging rate, the relative contents of unsaturated fatty acids (USFA) and saturated fatty acids (SFA), and USFA/SFA (U/S) were analyzed in juice sacs of HR pumelos stored for 90 d. In addition, combined with correlation analysis, we proposed a probable mechanism of lignin accumulation associated with energy metabolism and lipid peroxidation in postharvest pumelos during storage.

## 2. Materials and Methods

### 2.1. Plant Materials and Treatments

HR pumelos were harvested according to our previous work [6] from an orchard in Dapu county, Guangdong, China. Fruits at identical size and absent of mechanical injury were chosen and wrapped up by a 0.03 mm thick polyethylene film bag with one fruit in one bag at 25 °C and 85% relative humidity (RH). Six fruit were sampled at 0, 30, 60, and 90 d of storage. The juice sacs of fruit at each data point were taken and frozen in liquid nitrogen. All samples were prepared in three replicates and kept at −80 °C prior to use.

### 2.2. Determination of EC, NAD, and NADP Contents

ATP, ADP, and AMP levels were measured according to the previous study [6]. EC was computed with the formula: ([ATP] + 0.5 × [ADP])/([ATP] + [ADP] + [AMP]).

Detection of NAD and NADP contents were on the basis of the method of Lin et al. [46], one gram of powdered juice sacs of pumelo fruit was added with 4 mL of 0.1 M HCl, the mixture were heated at 95 °C for 5 min in an aqueous bath, cooled, and centrifuged at 10,000× *g* at 4 °C for 10 min. After neutralized with 0.1 M HCl, the supernatants were subjected to centrifugation at 10,000× *g* at 4 °C for 10 min. Then the supernatants were preserved on ice for further analysis. The enzymatic reaction was performed at 37 °C for 40 min with alcohol dehydrogenase and glucose-6-phosphate dehydrogenase as substrates for NAD and NADP detection, respectively. After dissolved in 1 mL 95% ethanol, the sample was detected at absorbance of 570 nm. The unit of the content of NAD and NADP was nM g^−1^ based upon fresh weight (FW) of juice sacs of pumelo fruit.

### 2.3. Determination of Activities of SDH, CCO, and F_0_F_1_-ATPase

Powdered juice sacs tissues (5 g) were employed for analyzing SDH, CCO, and F_0_F_1_-ATPase activities. The SDH and CCO activities were detected on the basis of the method of Li et al. [36]. Definition of one U for SDH activity was as the enzyme quantity that increased 0.05 at 600 nm absorbance in one min under the assay conditions of 1 g fresh tissue. Definition of one U for CCO activity was as the enzyme quantity that oxidized 1 nM cytochrome C in one min of 1 g fresh tissue. The activity of F_0_F_1_-ATPase was detected by using the F_0_F_1_-ATPase Assay Kit (Suzhou Grace Biotechnology Co., Suzhou, China) following the manufacturer’s instructions. Definition of one U for F_0_F_1_-ATPase activity was as the enzyme quantity that produced 1 nM NADPH in one min of 1 g fresh tissue. The SDH, CCO, and F_0_F_1_-ATPase activities were described as U g^−1^ FW.

### 2.4. Measurement of H^+^-ATPase and Ca^2+^-ATPase Activities

Five grams of juice sacs of pumelo fruit were taken to analyze the activities of H^+^-ATPase in mitochondria and Ca^2+^-ATPase in plant cell basing on the published method of Jin et al. [47] and method of Lin et al. [29], respectively. Definition of one U for H^+^-ATPase and Ca^2+^-ATPase activities were as the enzyme quantity that catalyzed the production of 1 μM phosphorus in one hour at 660 nm of 1 g fresh tissue. The H^+^-ATPase and Ca^2+^-ATPase activities were described as U g^-1^ FW.

### 2.5. Determination of Contents of H_2_O_2_, O_2_^−^ and MDA, and –OH Scavenging Rate

The H_2_O_2_ content, O_2_^−^ content, and −OH^.^ scavenging rate were measured using 5 g, 2 g, and 1 g of powdered juice sacs of pumelo fruit according to our previous work, respectively [48], the H_2_O_2_ content was described as μM g^−1^ FW, O_2_^−^ content was described as nM g^−1^ FW, and –OH scavenging rate was expressed as %. MDA content was determined according to our previous study [49] with 1 g of powdered juice sacs of pumelo fruit. The result was denoted as nM g^−1^ FW.

### 2.6. Determination of Relative Amounts of Fatty Acids and U/S

Relative amounts of fatty acids were determined according to our previous work [49]. Then, 1 g of powder was mixed with 5 mL petroleum ether and subsequently extracted via ultrasonic treatment at 50 °C for 30 min. The supernatant was dried through nitrogen stream and then blended with 6 mL n-hexane and 0.5 M KOH in methanol (*v*/*v* = 1:1), followed by incubation at 60 °C for 60 min in the oven. After centrifuging at 5000× *g* for 5 min, a 0.22 μm membrane was used to filter the supernatant. One μL filtrate was subjected to gas chromatograph (6890, Agilent Technologies Inc., Santa Clara, CA, USA) according to the method illustrated in the previous study [49]. Respective fatty acids were determined and detected basing on the comparison of the relative retention times and peak areas to standards. U/S was determined using the formula: (C_18:1_ + C_18:2_ + C_18:3_)/(C_16:0_ + C_18:0_).

### 2.7. Statistical Analysis

All data was displayed as the mean ± standard error (SE). Statistical analyses were performed using SPSS version 26 (SPSS, Inc., Chicago, IL, USA) via one-way analysis of variance (ANOVA) and Duncan’s test was used to test the significance of the difference (*p* < 0.05). The correlationships were performed using excel software (version 2013, Washington, DC, USA) via Pearson’s correlation analysis.

## 3. Results

### 3.1. Changes in EC, ATP/ADP, NAD, and NADP Contents

**Figure 1:**
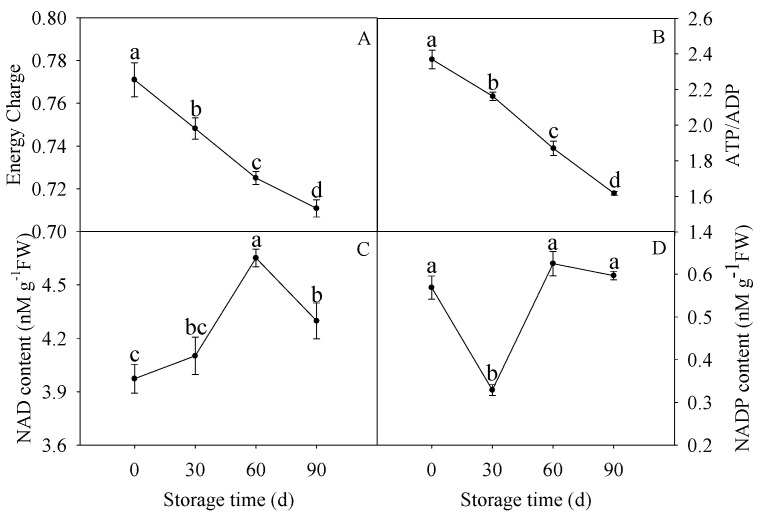
Changes in energy charge (EC) (**A**), ATP/ADP (**B**), nicotinamide adenine dinucleotide (NAD) (**C**), and nicotinamide adenine dinucleotide phosphate (NADP) content (**D**) in postharvest pumelos during storage at 25 °C. Each data point was shown as the mean ± standard error (SE). Different letters indicated significant difference (*p* < 0.05).

The EC (Figure 1A) and ATP/ADP (Figure 1B) decreased continuously from 0 d to 90 d. The NAD content displayed a slight rise, afterwards, increased significantly after 30 d of storage, and, then, decreased (Figure 1C). Moreover, the NADP content displayed a significant decline before 30 d of storage, afterwards, rise significantly after 30 d of storage and declined (Figure 1D).

### 3.2. Changes in SDH, CCO, and F_0_F_1_-ATPase Activities

**Figure 2:**
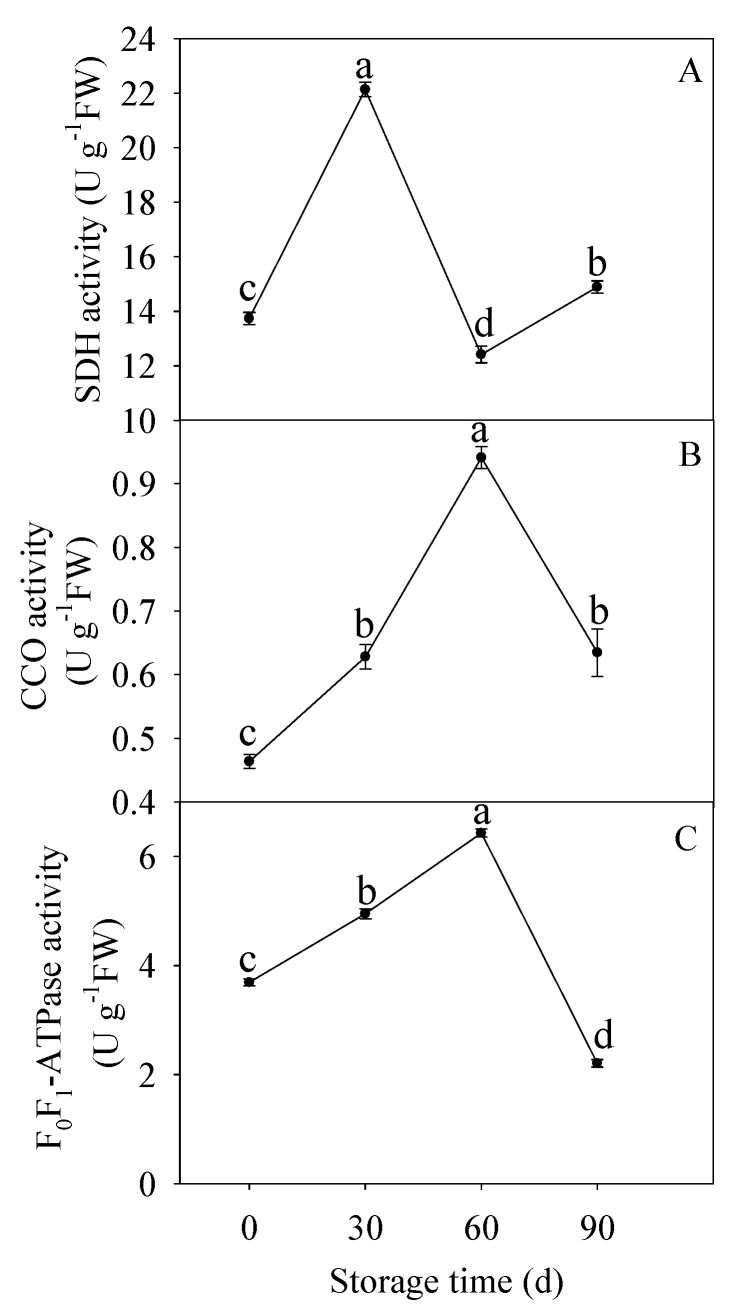
Changes in succinic dehydrogenase (SDH) (**A**), cytochrome *c* oxidase (CCO) (**B**), and F_0_F_1_-ATPase (**C**) activities in postharvest pumelos during storage at 25 °C. Each data point was shown as the mean ± standard error (SE). Different letters indicated significant difference (*p* < 0.05).

As shown in Figure 2A, the SDH activity increased significantly before 30 d of storage, thereafter, decreased markedly after 30 d of storage and changed slightly during the following storage. The CCO activity (Figure 2B) increased obviously before 60 d of storage, thereafter, significantly declined in the following storage. Moreover, Figure 2C illustrated that the F_0_F_1_-ATPase activity went up gradually before 60 d of storage, but declined obviously later.

### 3.3. Changes in H^+^-ATPase and Ca^2+^-ATPase Activities

**Figure 3:**
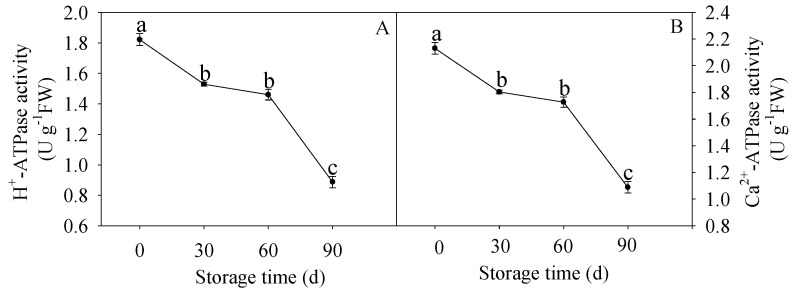
Changes in H^+^-ATPase (**A**) and Ca^2+^-ATPase (**B**) activities in postharvest pumelos during storage at 25 °C. Each data point was shown as the mean ± standard error (SE). Different letters indicated significant difference (*p* < 0.05).

The activity of H^+^-ATPase decreased significantly before 30 d of storage, thereafter, changed slightly and then a notable decline was displayed after 60 d of storage in Figure 3A. Interestingly, similar pattern of Ca^2+^-ATPase activity was demonstrated in our present work (Figure 3B).

### 3.4. Changes in Contents of H_2_O_2_, O_2_^−^, and MDA, and -OH Scavenging Rate

**Figure 4:**
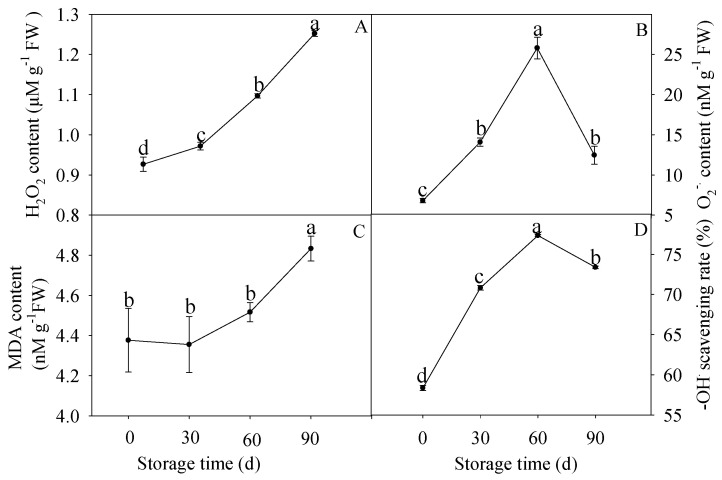
Changes in H_2_O_2_ content (**A**), O_2_^−^ content (**B**), malondialdehyde (MDA) content (**C**), and –OH scavenging rate (**D**) in postharvest pumelos during storage at 25 °C. Each data point was shown as the mean ± standard error (SE). Different letters indicated significant difference (*p* < 0.05).

As shown in Figure 4A, the H_2_O_2_ content increased slightly before 30 d of storage and then increased distinctly during the following storage. As for O_2_^−^ content, it increased significantly before of storage and dropped evidently later (Figure 4B). In addition, MDA content changed slightly, thereafter, increased significantly after 30 d of storage (Figure 4C). Differently, the –OH scavenging rate increased obviously before 60 d of storage and then decreased (Figure 4D).

### 3.5. Changes in Membrane Fatty Acids

**Figure 5:**
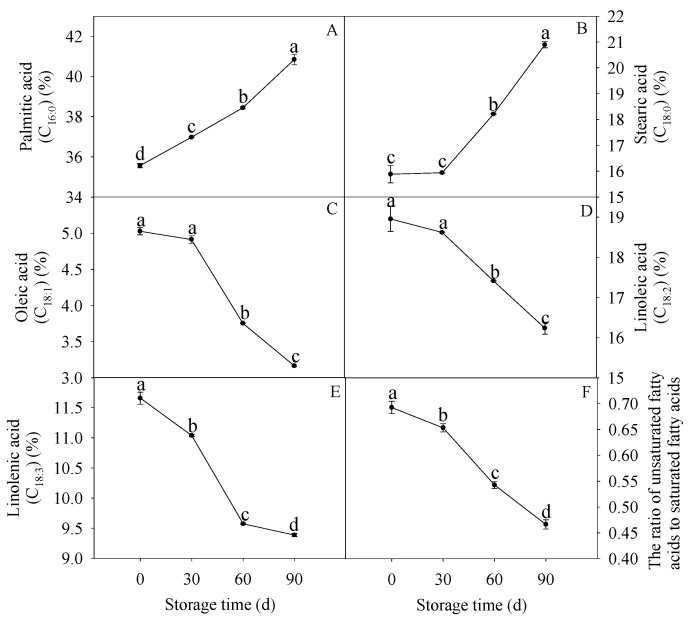
Changes in relative contents of palmitic acid (**A**), stearic acid (**B**), oleic acid (**C**), linoleic acid (**D**), and linolenic acid (**E**), and the relative contents of unsaturated fatty acids (USFA) to saturated fatty acids (SFA) (**F**) in postharvest pumelos during storage at 25 °C. Each data point was shown as the mean ± standard error (SE). Different letters indicated significant difference (*p* < 0.05).

As illustrated in Figure 5A, the relative amount of palmitic acid increased during the whole storage, while that of stearic acid maintained stablility firstly, and rose significantly after 30 d of storage (Figure 5B). As for unsaturated fatty acids, oleic acid and linoleic acid relative contents decreased slightly and subsequently declined after 30 d of storage (Figure 5C,D). Moreover, linolenic acid relative content decreased significantly firstly and declined slightly after 60 d of storage (Figure 5E). In accordance with the formation of SFA and the degradation of USFA, the U/S displayed a gradual decreasing tendency during the whole storage (Figure 5F).

## 4. Discussions

### 4.1. Lignin Accumulation Was Related to Energy Metabolism of Postharvest Pumelo Fruit

**Figure 6:**
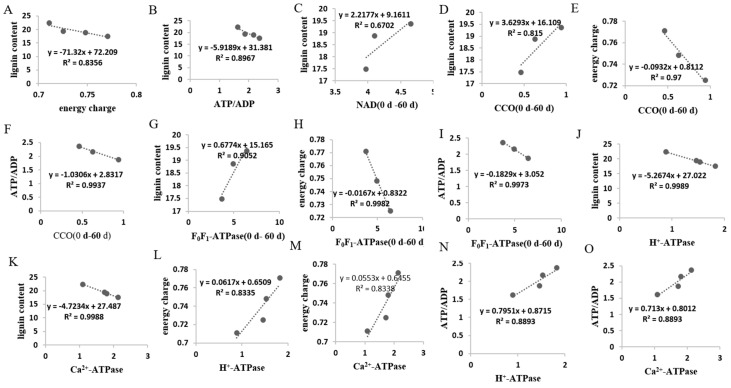
Correlationship between energy charge (**A**), ATP/ADP (**B**), NAD (0–60 d) (**C**), and CCO (0–60 d) (**D**) with lignin; between CCO (0–60 d) (**D**) with energy charge (**E**), and ATP/ADP (**F**); between F_0_F_1_-ATPase(0–60 d) with lignin (**G**), energy charge (**H**), and ATP/ADP (**I**); between H^+^-ATPase (**J**) and Ca^2+^-ATPase (**K**) with lignin; between H^+^-ATPase (**L**) and Ca^2+^-ATPase (**M**) with energy charge; and between H^+^-ATPase (**N**) and Ca^2+^-ATPase (**O**) with ATP/ADP, in juice sacs of “Hongroumiyou” (HR) pumelo fruit.

Our previous work displayed that the lignin content increased (Supplementary Figure S1) in HR pumelo fruit during the storage. In this work, EC and ATP/ADP decreased along with the whole storage of HR pumelo fruit (Figure 1A,B). As shown in Figure 6A and 6B, the linearity regression analysis suggested the lignin content was negatively correlated with the energy charge (R^2^ = 0.8356, y = −71.32x + 72.209) and ATP/ADP (R^2^ = 0.8967, y = −5.9189x + 31.381). Moreover, NAD and NADP, the primary types of pyridine nucleotide, were also associated with the energy level of harvested crops [12]. NAD and NADP principally functioned in predominating EMP-TCA cycle and stress response-related PPP respiratory pathway, respectively [50,51]. The NAD content increased in response to internal factor and environmental stimuli during postharvest fruit storage [52,53,54]. In this study, the NAD content in HR pumelos increased before 60 d storage (Figure 1C), indicating that the EMP-TCA cycle might be activated to cope with the ongoing stress in the earlier (0–30 d) and middle (30–60 d) storage stage. Meanwhile, NADP content dropped firstly but reached a peak at 60 d of storage and maintained high levels as storage extended (Figure 1D), suggesting that the stress response-related PPP respiratory pathway might be triggered in response to severe stress in the middle and later storage stage (30–90 d). In addition, correlation analysis revealed that the increased NAD content before 60 d of storage was positively correlated with lignin accumulation (R^2^ = 0.6702, y = 2.2177x + 9.1611) (Figure 6C). These findings suggested that energy deficit featured by declined EC, ATP/ADP, and NAD content indicated that lignin accumulation (Figure 1, Supplementary Figure S1) was correlative to energy status in postharvest HR pumelos.

Higher SDH and CCO activities are beneficial for the postharvest crops to cope with the internal and environmental stimuli factors [18]. However, it was reported that ultraviolet-C treatment reduced senescence development of pear fruit via reducing SDH and CCO activities [55]. Besides, The SDH and CCO activities showed a gradual decreasing tendency during broccoli postharvest storage [36,56], while a first increment reaching a peak and then a decline displayed during postharvest storage of pear [55] and litchi [57]. In our work, the activities of SDH and CCO showed similar patterns with postharvest pear fruit. Furthermore, the increased activities of CCO (Figure 2A) exhibited a positive correlation with the increased lignin accumulation (Supplementary Figure S1) before 60 d of storage (R^2^ = 0.815, y = 3.6293x + 16.109) (Figure 6D). Additionally, the increased activities of CCO (Figure 2A) exhibited a significant negative correlation with EC (R^2^ = 0.97, y = −0.0932x + 0.8112) (Figure 6E) and ATP/ADP (R^2^ = 0.9937, y = −1.0306x + 2.8317 (Figure 6F) from before 60 d of storage. These results showed that the enhanced CCO and SDH activities might result in energy deficit, which accelerated lignification process in postharvest HR pumelo fruit during the earlier and middle storage.

Similar with the CCO activities, the F_0_F_1_-ATPase activity increased firstly and then decreased after 60 d of storage (Figure 2C). As shown in Figure 6G, the correlation analysis showed that the F_0_F_1_-ATPase activity was in negative correlation with lignin content (R^2^ = 0.9052, y = −0.6774x + 15.165) before 60 d of storage. Furthermore, the F_0_F_1_-ATPase activity (Figure 2C) exhibited a significant negative correlation with the EC (R^2^ = 0.9982, y = −0.0167x + 0.8322) (Figure 6H) and ATP/ADP (R^2^ =0.9973, y = −0.1829x + 3.052) (Figure 6I) before 60 d of storage. H^+^-ATPase and Ca^2+^-ATPase are key enzymes in catalyzing ATP to release energy [58]. H^+^-ATPase is crucial for transmembrane electrochemical gradient construction and transmembrane electrochemical potential provision, suppression of its activity may lead to decline in the synthetic rate of ATP [58]. Ca^2+^-ATPase can utilize energy to transport Ca^2+^ from cytoplasm to mitochondria, maintaining the Ca^2+^ supply to ensure energy production, and to preserve the integrity of cell structure [7,59]. Abundant studies reported that insufficient energy and scanty ATPase activity could stimulate quality deterioration of postharvest fruits and vegetables such as banana [60], peach [61], litchi [9,24,57], broccoli [56], longan [7], and lotus [59]. In our work, the H^+^-ATPase and Ca^2+^-ATPase activities decreased gradually along with the postharvest storage (Figure 3A,B). These data demonstrated that decline of H^+^-ATPase and Ca^2+^-ATPase activities in juice sacs of HR pumelo might lead to ion imbalance and energy deficit, which might result in collapse of mitochondria, vacuole, and even the whole cell. Correlation analyses indicated that the decreased H^+^-ATPase and Ca^2+^-ATPase activities were remarkably negative correlated with the increased lignin content during the whole storage (R^2^ = 0.9989, y = −5.2674x + 27.022 and R^2^ = 0.9988, y = −4.7234x + 27.487, respectively) (Figure 6J,K).

Moreover, the decreased H^+^-ATPase and Ca^2+^-ATPase activities (Figure 3A,B) displayed significant positive correlations with the decreased EC (R^2−^= 0.8335, y = 0.0617x + 0.6509 and R^2^ = 0.8338, y = 0.0553x + 0.6455, respectively) (Figure 6L,M) and ATP/ADP (R^2^ = 0.8893, y = 0.7915x + 0.8715 and R^2^ = 0.8893, y = 0.713x + 0.8012, respectively) (Figure 1A,B) (Figure 6N,O) during the whole storage stage. These results revealed unexpected role of ATPase in energy metabolism, which gave evident credence for the involvement of these enzymes in the lignin accumulation of HR pumelo fruit during postharvest storage.

### 4.2. Lignin Accumulation Was Related to Lipid Peroxidation of Postharvest Pumelo Fruit

**Figure 7:**
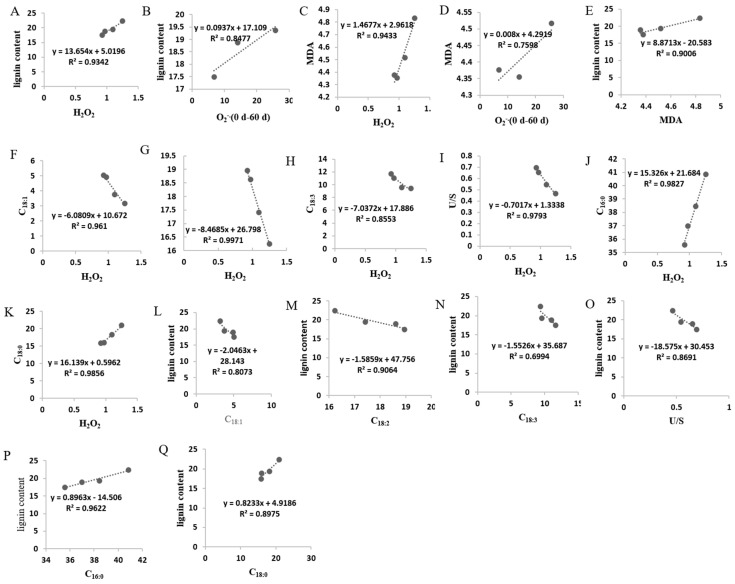
Correlationship between H_2_O_2_ (**A**) and O_2_^−^ (0–60 d) (**B**) with lignin; between H_2_O_2_ (**C**) and O_2_^−^ (0–60 d) (**D**) with MDA; between MDA with lignin (**E**); between H_2_O_2_ with C_18:1_ (**F**), C_18:2_ (**G**), C_18:3_ (**H**), USFA/SFA (U/S) (**I**), C_16:0_ (**J**), and C_18:0_ (**K**); and between C_18:1_ (**L**), C_18:2_ (**M**), C_18:3_ (**N**), U/S (**O**), C_16:0_ (**P**), and C_18:0_ (**Q**) with lignin, in juice sacs of HR pomelo fruit.

ROS molecules (such as H_2_O_2_, O_2_^−^, or –OH) are mainly generated from oxidative phosphorylation in the respiration cycle. ROS was reported to accumulate in various postharvest crops, such as grape [62], peach [63], longan [37], litchi [64], and pumelo [65], during storage. Excessive H_2_O_2_ and O_2_^−^ accelerate the peroxidation of membrane lipids, which might result in MDA accumulation and lead to fruit senescence [66]. Previous reports showed that continuously accumulated ROS-related characteristics were accompanied with the increment of lignin content in asparagus [67], sugarcane [68], and bamboo shoots [45]. In the current study, the H_2_O_2_ content increased as storage time processed (Figure 4A). Correlationship analysis displayed that the increased H_2_O_2_ content was positively related with increased lignin content significantly (R^2^ = 0.9342, y = 13.654x + 5.0196) (Figure 7A), which is in accordance with that H_2_O_2_ plays a catalytic role in lignification via acting as substrates of peroxidases during the oxidative polymerization of monoligols to lignin process [43]. The content of O_2_^−^ went up firstly and then decreased after 60 d of storage (Figure 4B). Correlationship analysis displayed that the increased O_2_^−^ content was positively correlated with increased lignin content before 60 d of storage significantly (R^2^ = 0.8477, y = 0.0937x + 17.109) (Figure 7B). Meanwhile, MDA content increased with the extension of storage (Figure 4C). It is worth noting that significant positive relation was demonstrated between H_2_O_2_ content and MDA content during the whole storage (R^2^ = 0.9433, y = 1.4677x + 2.9618) (Figure 7C), in addition, the increased O_2_^−^ content was positively related with MDA content before 60 d of storage (R^2^ = 0.7598, y = 0.008x + 4.2919) (Figure 7D), implying that the excessive H_2_O_2_ and O_2_^−^ content promoted peroxidation of cell membrane lipids, which resulted in MDA accumulation, and lipid membrane was consequently damaged and senescence process of pumelo fruit was accelerated. Furthermore, correlationship analysis displayed that MDA content was significantly positively correlated with increased lignin content (R^2^ = 0.9006, y = 8.8713x − 20.583) (Figure 7E). Interestingly, the scavenging rate of –OH showed a gradual increasing tendency in the earlier and middle storage period and a subsequent decline in the later storage period (Figure 4D). In our previous study, the activities of APX and GPX played a role in scavenging the ROS in postharvest pumelo fruit [69]. Furthermore, there was a sharp increment of lignin content in HR pumelo fruit in the later storage stage (Supplementary Figure S1), which was in accordance with dysfunction of the ROS scavenging system during this stage (Figure 4). These results indicated that the ripening of pumelo fruit is accompanied by a progressive increase in oxidative/peroxidative stress which prompted ROS scavenging system but not until later stages of ripening, which lead to over-accumulation of ROS at later stages of fruit ripening resulting in tissue structure breakdown. The monolignol/lignin precursors in the cell must cross the cell membrane to the cell wall to synthesis lignin and membrane translocation rates of the monolignol/lignin precursors are regulated by compound delivery and utilization rates and membrane concentration gradients [42], suggesting that membrane integrity loss will result in accelerated translocation rates, eventually stimulating lignin synthesis. Taken collectively, these data demonstrated that lignin accumulation was closely related with excessive ROS accumulation in postharvest HR pumelos.

Cell membrane system is crucial in the normal physiological metabolism of fruit [70]. Lipids are important structural blocks of cell membrane and variation in compositions of membrane lipids might incur membrane properties alteration, cellular compartmentalization loss, and membrane permeability increment [71,72]. USFA are critical for plant adaptation to various stresses in that they endow unsaturation and fluidity to cell membranes [73]. Alteration of USFA influences fluidity and integrity of cell membrane, and this issue is especially prominent in fresh harvested crops, where a decline in USFA amount but a rise in SFA content of the crops were observed during storage. Postharvest treatment can alleviate quality deterioration of postharvest crops, such as longan, pear, lotus, and kiwifruit, via maintaining higher USFA levels, decreasing SFA production [60,72,74,75]. Similarly, the SFA relative contents increased but that of USFA declined during the storage period in our work. Meanwhile, the U/S reduced along with the storage process (Figure 5F). Significantly negative correlations were found between the relative contents of USFA and H_2_O_2_ content (Figure 7F–H) and between U/S and H_2_O_2_ content (Figure 7I). However, remarkably positive correlations were displayed between the relative content of SFA and H_2_O_2_ content (Figure 7J,K), and between MDA content and H_2_O_2_ content (Figure 7C). Moreover, there were negative correlations between the relative content of USFA and lignin content (Figure 7L–N), and between U/S and lignin content in pumelo fruit along with the storage period (Figure 7O). However, the relative contents of SFA were significantly positive correlated with lignin content (Figure 7P,Q). Taken collectively, these data implied that the ROS accumulation mediated alteration from USFA to SFA, resulting in lipid peroxidation and membrane integrity loss, which promoted the lignin accumulation in HR pumelo fruit during postharvest storage.

## 5. Conclusions

**Figure 8:**
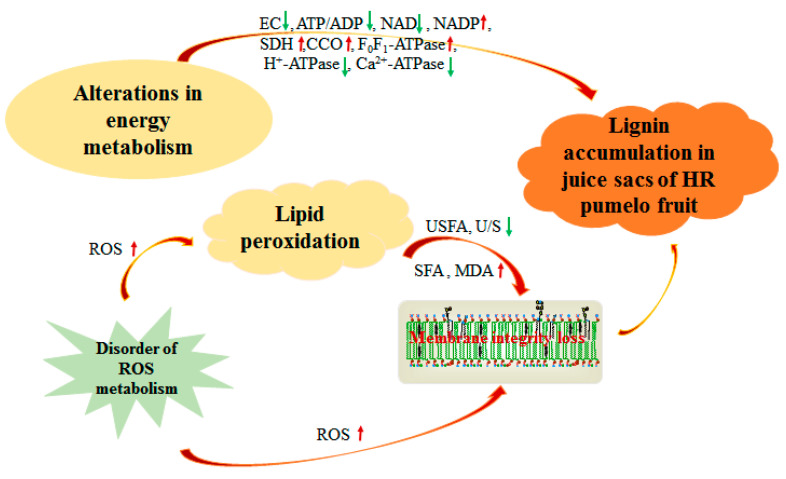
The probable mechanism of lignin accumulation affected by alterations in energy metabolism and membrane lipid metabolism in juice sacs of postharvest HR pumelos during storage period.

This study explored lignin accumulation in postharvest pumelos in association with energy metabolism and lipid peroxidation. In brief, the results displayed that the lignin accumulation in HR pumelos was closely associated with energy metabolism, which was mainly attributed to declined EC and ATP/ADP, increased SDH activity before 30 d, and increased CCO and F_0_F_1_-ATPase activities before 60 d; but decreased H^+^-ATPase and Ca^2+^-ATPase activities. In addition, increased ROS level and MDA content induced the oxidation of USFA to SFA, resulting in lipid peroxidation and membrane integrity loss. These together may promote the lignin accumulation in postharvest HR pumelos during storage period. The probable mechanism of lignin accumulation in postharvest HR pumelos in association with energy metabolism and lipid peroxidation was shown in Figure 8.

## Supplementary Materials

The following are available online at https://www.mdpi.com/2077-0375/10/10/269/s1, Figure S1: Changes in lignin content in juice sacs of HR pomelo fruit during postharvest storage.
