# Supplementary material for: The Involvement of Energy Metabolism and Lipid Peroxidation in Lignin Accumulation of Postharvest Pumelos
Source: Membranes (Basel). 2020 Sep 30;10(10):269. doi: 10.3390/membranes10100269 (PMC7599556; doi:10.3390/membranes10100269)
Supplement: Supplementary file 1 [file membranes-10-00269-s001.pdf]

membranes

Supplementary Materials: The Involvement of Energy Metabolism and Lipid Peroxidation in Lignin Accumulation of Postharvest Pumelos

Huiling Yan [2,3], Junjia Chen [1] and Juan Liu [1,*]

[1] Guangdong Engineering Lab of High Value Utilization of Biomass, Institute of Bioengineering, Guangdong Academy of Sciences, Guangzhou 510316, China; gzcsircjj@163.com
[2] Key Laboratory of Plant Resources Conservation and Sustainable Utilization, Guangdong Provincial Key Laboratory of Applied Botany, South China Botanical Garden, Chinese Academy of Sciences, Guangzhou 510650, China; hlingyan@scbg.ac.cn
[3] University of Chinese Academy of Sciences, Beijing 100049, China
* Correspondence: ljane0505@126.com

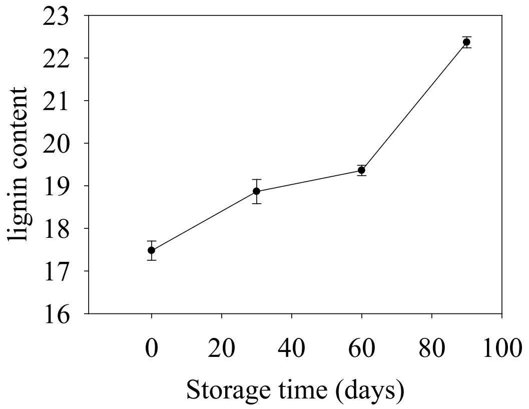

Figure S1. Changes in lignin content in juice sacs of HR pomelo fruit during postharvest storage. Data had been published in Biomolecules (Liu, Huang, Kang, Liang, & Chen, 2019, https://doi.org/10.3390/biom9110701).
